# Genomic Epidemiology of Carbapenem-Resistant *Acinetobacter baumannii* Isolated from Patients Admitted to Intensive Care Units in Network Hospitals in Southern Thailand

**DOI:** 10.3390/antibiotics15020133

**Published:** 2026-01-28

**Authors:** Arnon Chukamnerd, Komwit Surachat, Rattanaruji Pomwised, Prasit Palittapongarnpim, Kamonnut Singkhamanan, Sarunyou Chusri

**Affiliations:** 1 Division of Infectious Disease, Department of Internal Medicine, Faculty of Medicine, Prince of Songkla University, Songkhla 90110, Thailand; arnonchukamnerd@hotmail.com; 2 Department of Biomedical Sciences and Biomedical Engineering, Faculty of Medicine, Prince of Songkla University, Songkhla 90110, Thailand; komwit.s@psu.ac.th (K.S.); skamonnu@medicine.psu.ac.th (K.S.); 3 Division of Biological Science, Faculty of Science, Prince of Songkla University, Songkhla 90110, Thailand; rattanaruji.p@psu.ac.th; 4 Pornchai Matangkasombut Center for Microbial Genomics, Department of Microbiology, Faculty of Science, Mahidol University, Bangkok 10400, Thailand; prasit.pal@mahidol.ac.th

**Keywords:** carbapenem-resistant *Acinetobacter baumannii*, whole-genome sequencing, genomic epidemiology, lower southern Thailand, high-risk ST2

## Abstract

**Background/Objectives**: Carbapenem-resistant *Acinetobacter baumannii* (CRAB) is classified as an urgent-threat pathogen because of its resistance to nearly all available antibiotics, resulting in high morbidity and mortality rates. However, data on the molecular epidemiology of CRAB isolates in southern Thailand are limited. This study aimed to investigate the genomic epidemiology of CRAB isolates within a hospital network in lower southern Thailand. **Methods**: Whole-genome sequencing data of CRAB clinical isolates (*n* = 224) were obtained from a previous study. Additional isolates (*n* = 70) were included, for which genomic DNA was extracted and sequenced. In total, 294 isolates were collected from patients across seven hospitals in southern Thailand between 2019 and 2020. Their genomes were analyzed using several bioinformatic tools. **Results**: A high proportion of isolates were obtained from sputum samples of patients with CRAB infection or colonization. Sequence type (ST) 2 was the most frequent ST and was classified in the quadrant with high resistance and virulence. The Sankey diagram showed that ST2 was the dominant and most versatile CRAB lineage circulating across major hospitals, commonly associated with pneumonia, and that diverse resistance genes and plasmid combinations were dominated by *bla*_OXA-23_. The core single-nucleotide polymorphism (SNP)-based phylogenetic tree revealed clades A1 (ST215), A2 (multiple STs), and B (ST2). Bloodstream, skin, and soft tissue infections were predominantly observed in clade B. **Conclusions**: Our analysis revealed widespread circulation of a high-risk ST2 CRAB lineage with enhanced resistance and virulence across hospital networks in the studied region, highlighting the importance of genomics-informed surveillance for controlling CRAB dissemination.

## 1. Introduction

*Acinetobacter baumannii* (*A. baumannii*) is listed as one of the ESKAPE pathogens (*Enterococcus faecium*, *Staphylococcus aureus*, *Klebsiella pneumoniae*, *A. baumannii*, *Pseudomonas aeruginosa*, and *Enterobacter* species) and is a highly virulent, multidrug-resistant bacterium that is a major cause of hospital-acquired infections [1]. Its remarkable ability to persist in hospital environments and rapidly acquire resistance genes has led to the emergence of strains that are resistant to multiple antibiotics [2]. Carbapenem-resistant *Acinetobacter baumannii* (CRAB) is considered an urgent-threat pathogen because of its resistance to virtually all antimicrobial agents, resulting in extensively drug-resistant and difficult-to-treat infections [3]. The primary mechanisms of carbapenem resistance in *A. baumannii* involve the production of carbapenemase enzymes, most notably OXA-23 oxacillinases, along with the overexpression of multidrug efflux pumps and loss of outer membrane porin function [4]. Although these mechanisms are rarely specific to a single drug, they provide cross-resistance to a broad spectrum of β-lactams. CRAB isolates usually harbor additional genetic determinants that confer resistance to other antimicrobial classes, including aminoglycosides, tetracyclines, and fluoroquinolones [5]. In addition to its resistant phenotype, the pathogenicity of *A. baumannii* is bolstered by several key virulence factors, including chaperone-usher fimbriae for surface attachment, biofilm-associated proteins for environmental persistence, and quorum-sensing systems that coordinate population-level gene expression [6,7].

To track an infectious disease outbreak and study its genomic characteristics, many studies have previously reported the whole-genome sequencing (WGS) data of CRAB isolates from Europe, Asia, and South America [8,9,10,11,12,13,14]. A consistent finding across these reports is the dominance of a few highly successful sequence types (STs), such as ST2 (part of the Global Clone 2 (GC2)/International Clone 2 (IC2)), which was prevalent in isolates from Europe, Thailand, and Vietnam. Other significant lineages, such as ST1, ST492 (Europe), ST191 (South Korea), and ST195/ST208 (Malaysia), also contributed to resistance. This pattern confirms that local CRAB epidemiology is typically dominated by the successful clonal expansion of specific lineages that have acquired resistance. Notably, the underlying mechanism was overwhelmingly centered on the *bla*_OXA-23_ gene—identified as the most prevalent carbapenemase detected in isolates from Brazil, South Korea, and Southeast Asia—suggesting its widespread acquisition or superior fitness within these dominant clones. The role of mobile genetic elements (MGEs) in facilitating the acquisition and mobility of resistance genes is a critical aspect of genomic data. Specifically, the determinants of resistance are consistently found to be closely associated with insertion sequences (IS), which act as strong promoters or facilitate transposition. IS*Aba1* and IS*Aba125* were found upstream of *bla*_OXA-23_ and *bla*_NDM-1_, respectively. These genes are frequently carried on various plasmids, with the repAci1 plasmid lineage observed at a high frequency in Thailand. Less prevalent genes—such as *bla*_NDM-1_—are typically harbored by minor, genetically distinct STs, suggesting independent introduction or importation events and reflecting a dissemination dynamic different from the endemic, clonally driven spread of *bla*_OXA-23_. These international studies have demonstrated a global convergence of successful CRAB clones and resistance genes, emphasizing the need for continuous surveillance to manage this public health threat.

As the transmission dynamics and clonal makeup of CRAB can vary significantly by region, localized data is of paramount importance. To date, a comprehensive molecular surveillance of CRAB in southern Thailand has not been conducted. Therefore, we aimed to investigate the genomic epidemiology of CRAB clinical isolates from seven hospitals within a hospital network in southern Thailand using short-read WGS data.

## 2. Results

### 2.1. Surveillance Period and Clinical Characteristics

**Figure 1:**
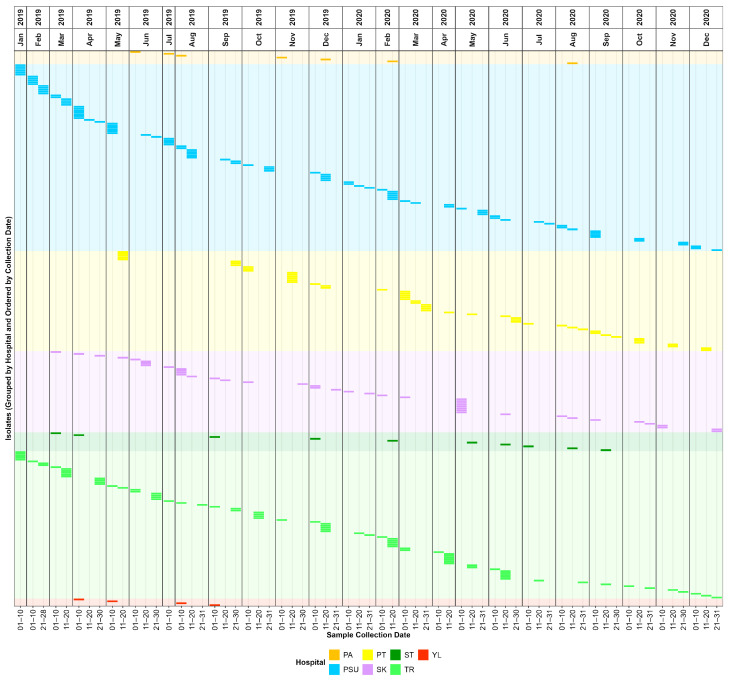
Timeline of sample collection, grouped by hospital and ordered by collection date. The timeline was generated using the ggplot2 package in R. PA, Pattani Hospital; PSU, Songklanagarind Hospital; PT, Phatthalung Hospital; SK, Songkhla Hospital; ST, Satun Hospital; TR, Trang Hospital; YL, Yala Hospital.

Figure 1 and Table S1 present the sample collection timeline. A total of 294 CRAB clinical isolates were successfully recovered from seven participating hospitals in southern Thailand during a 24-month surveillance period from January 2019 to December 2020. The collection rate was slightly higher in 2019 (*n* = 159, 54.08%) than in 2020 (*n* = 135, 45.92%), representing a diverse regional snapshot of CRAB prevalence. Visual analysis of the isolation timeline confirmed that CRAB was endemic across most participating facilities, with isolates recovered in nearly every month of the 2-year study, particularly in Songklanagarind, Trang, and Songkhla Hospitals (Figure 1). While the overall trend showed a slight decrease in 2020, Phatthalung Hospital experienced a notable increase in isolation frequency, rising from 20 isolates in 2019 to 33 isolates in 2020. In contrast, all four isolates from Yala Hospital were exclusively identified in 2019, and no further isolates were recovered in 2020. Despite the continuous surveillance maintained throughout the study period, these fluctuations likely reflect a combination of true epidemiological shifts and variations in sampling intensity or logistical constraints.

Table 1 and Table S1 present the clinical characteristics of the 294 patients. Patient demographics showed an age range of 5–96 years, with a median age of 52 years. More than half of the patients were male (58.50%). The duration of hospital stay ranged from 2 to 62 days, with a median of 12 days and an interquartile range (IQR) of 9–19 days. In total, 241 patients (81.97%) had underlying conditions, such as diabetes mellitus, hypertension, dyslipidaemia, chronic kidney disease, cerebrovascular disease, coronary artery disease, and/or pulmonary disease. The median Commodity Channel Index (CCI) score was seven (IQR: 5–8). Almost all patients (99.32%) were exposed to at least one antibiotic within the previous three months. The most commonly used prior antibiotics were meropenem (98.98%), piperacillin/tazobactam (55.78%), and imipenem (47.62%). Importantly, 262 patients (89.12%) were exposed to at least one carbapenem (meropenem, imipenem, and/or ertapenem). Additionally, the highest frequency of isolates was obtained from sputum (65.99%), followed by urine (10.88%), blood (10.20%), pus (9.18%), ascites (2.04%), and pleural effusion (1.70%). Pneumonia was the most frequent clinical diagnosis (41.50%), followed by colonization, which was present in 29.93% of the cohort. The median Acute Physiology and Chronic Health Evaluation II score was 16 (IQR: 12–16).

### 2.2. Antimicrobial Susceptibility Profiles

Table S2 and Table 2 present the results of the antimicrobial susceptibility testing of the 294 CRAB isolates. Almost all the studied CRAB isolates were resistant to imipenem, meropenem, ceftazidime, cefoperazone/sulbactam, amikacin, gentamicin, ciprofloxacin, levofloxacin, and trimethoprim/sulfamethoxazole (co-trimoxazole). However, a high proportion of isolates remained susceptible to colistin and tigecycline. Similar antimicrobial susceptibility patterns were observed among isolates from each of the participating hospitals. Among the carbapenem-resistant isolates, 273 (92.86%) were resistant to both imipenem and meropenem, whereas 21 (7.14%) were resistant to only one of these two agents.

### 2.3. Genome Assembly Quality

Table S3 presents the QUAST reports and accession numbers of all studied CRAB genomes. A total of 22–193 contigs were identified, with lengths and percent GC content ranging from 3,774,344 to 4,319,283 base pairs (bp) and 38.71% to 39.17%, respectively. The complete and single-copy sequences of all the assembled genomes were over 96%, with relatively low fragmentation percentages. Neither duplicated nor missing sequences were found.

### 2.4. Identification of Sequence Types (STs), Virulence-Associated Genes (VAGs), Antimicrobial Resistance Genes (ARGs), and Plasmid Marker Genes (PMGs)

**Figure 2:**
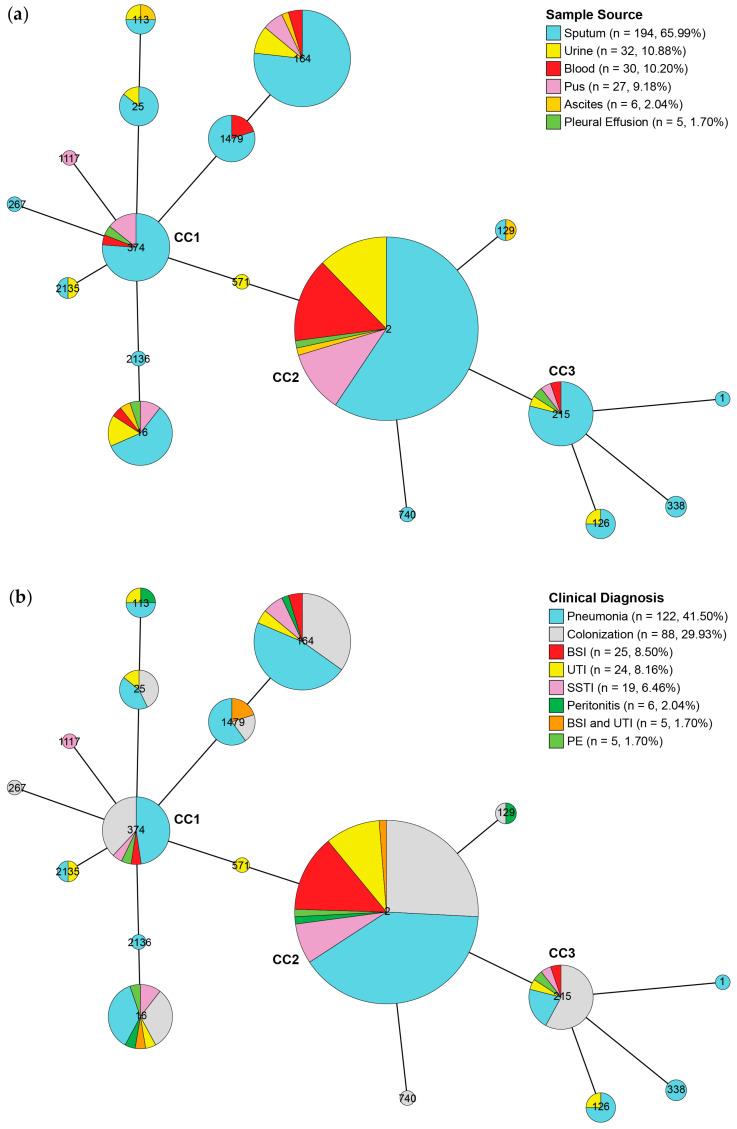
Minimum spanning tree (MST) of all carbapenem-resistant *Acinetobacter baumannii* (CRAB) clinical isolates based on multilocus sequence typing (MLST) data, visualized with sample sources (**a**) and clinical diagnoses (**b**). The MST was generated using GrapeTree v1.5.0. CC, clonal complex; BSI, bloodstream infection; UTI, urinary tract infection; SSTI, skin/soft tissue infection; PE, pleural effusion.

Sequence type (ST) 2 (*n* = 155, 52.72%) was the most frequently identified ST among all studied CRAB isolates, followed by ST164 (*n* = 43, 14.63%), ST374 (*n* = 21, 7.14%), and others (Table S4). Among the most dominant ST2 isolates, 64 (41.29%) were collected from patients admitted to the Trang Hospital. The PSU114 and PSU262 isolates belonged to ST2135, whereas the PSU043 isolate belonged to ST2136. Both ST2135 and ST2136 are novel STs defined in this study. As illustrated in Figure 2, the minimum spanning tree (MST) constructed from multilocus sequence typing (MLST) data identified three clonal complexes (CCs). ST374 occupied a central position within the largest CC, designated as CC1, and was connected to ST16, ST25, ST164, ST267, ST1479, ST113, ST1117, ST2135, and ST2136. The most frequent ST, ST2, was grouped with ST129, ST517, and ST740 within CC2, with ST517 serving as a bridge between CC1 and CC2. CC2 was directly linked to CC3, including ST1, ST126, and ST338. Although more than half of the isolates collected from the sputum were associated with pneumonia, many were identified as colonizing isolates.

**Figure 3:**
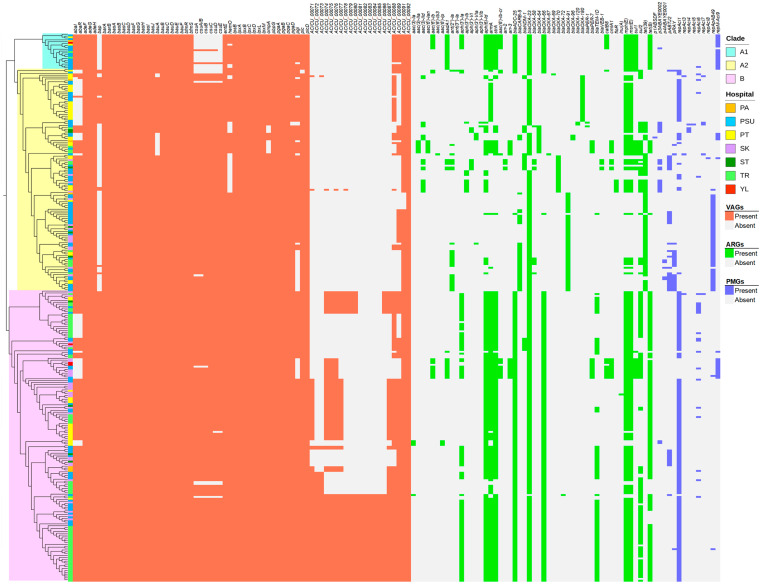
Patterns of virulence-associated genes (VAGs), antimicrobial resistance genes (ARGs), and plasmid marker genes (PMGs) in the core single-nucleotide polymorphism (SNP)-based phylogenetic tree of all carbapenem-resistant *Acinetobacter baumannii* (CRAB) clinical isolates. The heatmap and tree were generated using Microsoft Excel and FastTree v2.1.11, respectively.

Figure 3 shows the presence of virulence-associated genes (VAGs) among all studied CRAB isolates. All CRAB isolates harbored various VAGs. Efflux pump genes (*adeF*, *adeG*, and *adeH*), penicillin-binding protein (PBP) gene (*pbpG*), and genes involved in biofilm formation and regulation (*bap*, *pgaA*, *pgaB*, *pgaC*, *bfmR*, and *bfmS*) were present in all the isolates. All isolates carried complete sets of siderophore biosynthesis and transport genes, namely, the *basA–basJ* and *bauB–bauF* clusters, along with *entE*. Lipopolysaccharide and lipid A biosynthesis genes (*lpsB*, *lpxA*, *lpxB*, *lpxC*, *lpxD*, *lpxL*, and *lpxM*) were detected consistently. Other virulence determinants, including *barA*, *barB*, and *plcD*, were identified in all the genomes. Figure 3 shows the presence of acquired antimicrobial resistance genes (ARGs) in all studied CRAB isolates. Our findings showed that 266 (90.48%) and 8 (2.72%) isolates carried the *bla*_OXA-23_ and *bla*_NDM-1_ genes in the investigation of carbapenem resistance. Co-harboring of *bla*_OXA-23_ + *bla*_NDM-1_, *bla*_NDM-1_ + *bla*_OXA-58_, and *bla*_OXA-23_ + *bla*_OXA-58_ was also observed in eight (2.72%), six (2.04%), and five (1.70%) isolates, respectively. However, none of these carbapenemase genes were found in the PSU270 isolate. Other ARGs were also detected in the studied isolates, including the genes conferring resistance to other β-lactams (e.g., *bla*_ADC-25_ and *bla*_TEM-1D_), macrolides (e.g., *mphE* and *msrE*), aminoglycosides (e.g., *aph(6)-Id* and *armA*), tetracyclines (e.g., *tet(39)* and *tet(B)*), sulfonamide (e.g., *sul1* and *sul2*), chloramphenicol (e.g., *cmlA1*), rifampicin (e.g., *arr-2* and *arr-3*), and lincosamide (e.g., *lnu(A)*). For plasmid prediction, the number of identified plasmid marker genes (PMGs) ranged from one to four (Figure 3). The *repAci1* plasmid replicase gene was detected in the highest proportion of the isolates. Combined analysis of VAGs, ARGs, and PMGs revealed a relatively uniform distribution within each phylogenetic clade; however, distinct differences were observed among the clades.

### 2.5. Association Between Resistance and Virulence Across the STs

**Figure 4:**
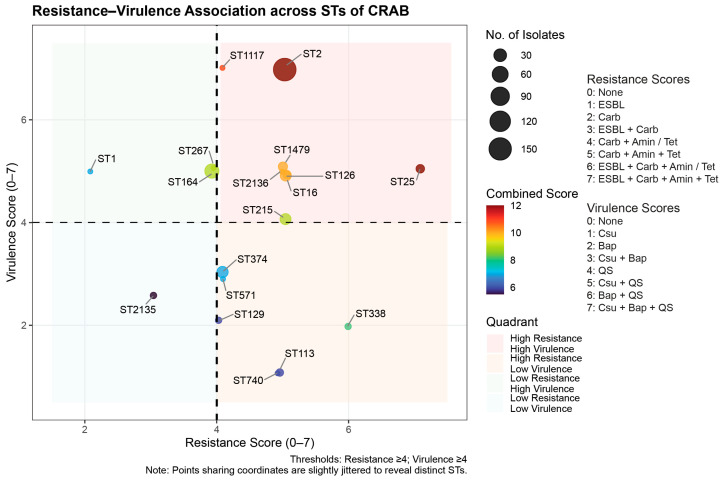
Association between resistance and virulence across sequence types (STs) in all carbapenem-resistant *Acinetobacter baumannii* (CRAB) clinical isolates. The figure was generated using the ggplot2 package in R. ESBL, extended-spectrum β-lactamase gene(s); Carb, carbapenemase gene(s); Amin, aminoglycoside resistance gene(s); Tet, tetracycline resistance gene(s); Csu, chaperone-usher fimbriae gene(s); Bap, biofilm-associated protein gene(s); QS, quorum-sensing gene(s).

The presence of ARGs (extended-spectrum β-lactamase (ESBL), carbapenemase, aminoglycoside resistance, and tetracycline resistance genes) and VAGs (chaperone-usher fimbriae, biofilm-associated protein, and quorum-sensing genes) was incorporated into the scoring framework to elucidate the relationship between antimicrobial resistance and virulence determinants. Resistance and virulence scores were combined and evaluated across the distribution of STs identified in all CRAB clinical isolates. As illustrated in Figure 4, the ST2 isolates, along with the clusters of ST25, ST1117, ST16, ST126, ST1749, ST2136, and ST215 isolates, were plotted in a distinct quadrant, representing high resistance and virulence traits. Notably, ST2 and ST1117 isolates displayed the highest virulence (approximately 7) together with high resistance (approximately 5), whereas ST25 isolates showed the opposite pattern, with the highest resistance (approximately 7) and high virulence (approximately 5). Only ST2135 isolates showed low resistance and virulence characteristics. ST338, ST571, ST113, ST740, ST374, and ST129 isolates were grouped in the quadrants of high resistance and low virulence traits. Meanwhile, ST1, ST164, and ST267 isolates were categorized in the quadrant of low-resistance and high-virulence characteristics.

### 2.6. Association Among Hospitals, Clinical Diagnoses, STs, Carbapenem Resistance-Associated Genes, and Predicted PMGs

**Figure 5:**
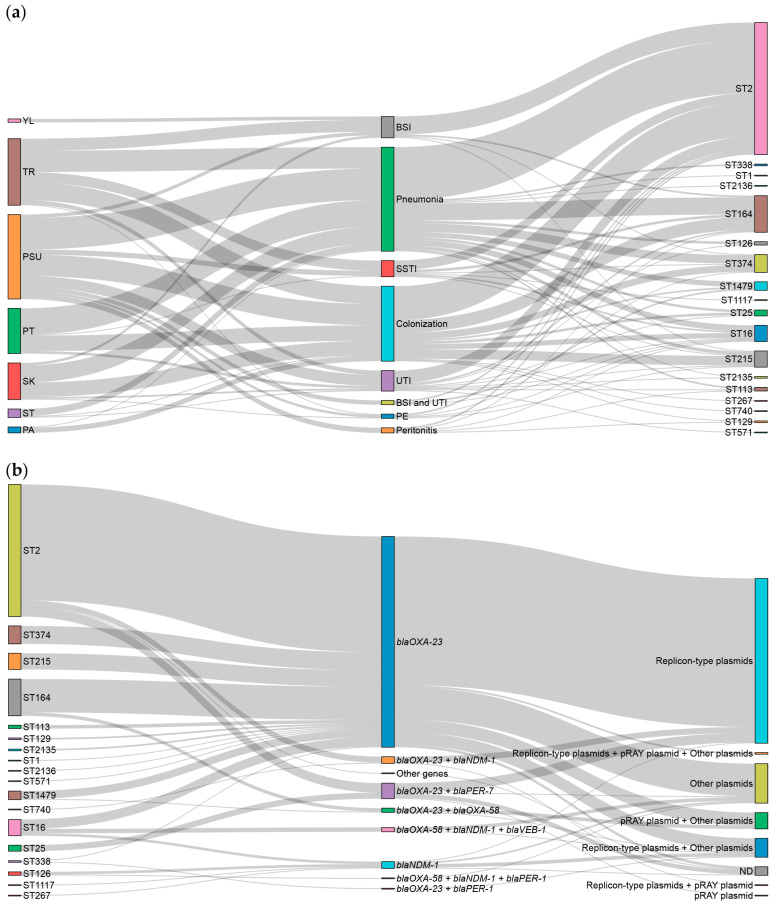
Sankey diagrams showing (**a**) associations among hospitals, clinical diagnosis, and sequence types, and (**b**) associations among sequence types, carbapenem resistance-associated genes, and predicted plasmid marker genes. The diagram was generated using the plotly package in R. PA, Pattani Hospital; PSU, Songklanagarind Hospital; PT, Phatthalung Hospital; SK, Songkhla Hospital; ST, Satun Hospital; TR, Trang Hospital; YL, Yala Hospital; BSI, bloodstream infection; UTI, urinary tract infection; SSTI, skin/soft tissue infection; PE, pleural effusion. Replicon-type plasmids were repAci1, repAci3, repAci4, repAci5, repAci6, repAci7, repAci8, repApAB49, and repM-Aci9. Other plasmids included p1ABSDF, p3ABAYE0002, p4ABAYE0001, and pABTJ2.

The Sankey diagram (Figure 5) illustrates the distribution and co-localization of hospitals, clinical diagnoses, STs, carbapenem resistance-associated genes, and predicted PMGs. Figure 5a highlights a complex epidemiological landscape in which Songklanagarind, Trang, and Phatthalung Hospitals served as major hubs for a diverse range of CRAB lineages. A central finding of this study was the overwhelming dominance and versatility of ST2 isolates (*n* = 155). Although many STs appear specialized or restricted to colonization, ST2 emerged as a versatile lineage capable of causing many infection types. Furthermore, the diversity observed within the colonization category feeds into nearly every listed ST. Remarkably, most of the studied isolates were associated with CRAB infections; however, nearly all isolates from Pattani Hospital (6/7, 85.71%) were recovered from patients with CRAB colonization. All isolates from patients with peritonitis and those with concomitant bloodstream infection (BSI) and urinary tract infection (UTI) were exclusively identified at Songklanagarind Hospital. Although UTI isolates were primarily found at Songklanagarind Hospital, isolates from BSI and skin/soft tissue infections (SSTI) were predominantly observed at Trang Hospital. ST2 isolates were primarily associated with pneumonia (62/155, 40%), followed by colonization (40/155, 25.81%). Most ST215 isolates (11/19, 57.89%), as well as all ST267 and ST740 isolates, were associated with CRAB colonization.

As shown in Figure 5b, ST2 and ST164 were the most dominant STs contributing to the observed resistant genotype, characterized by the thickest flow bands originating from these nodes. Most isolates from all STs converged heavily on a single carbapenemase gene, *bla*_OXA-23_, which is the most prevalent resistance determinant in this population. A combination of *bla*_OXA-23_ + *bla*_OXA-58_ was observed in some ST164 isolates, while combinations of *bla*_OXA-23_ + *bla*_NDM-1_ and *bla*_OXA-23_ + *bla*_PER-7_ were observed in some ST2 isolates. Notably, *bla*_NDM-1_ was exclusively found in isolates from minor STs (ST16, ST126, ST267, and ST1117). Although minor STs, including ST16, ST25, ST126, and ST338, contributed to a small fraction of isolates, they exhibited greater diversity in the combination of carbapenemase and ESBL genes. Transfer and mobilization of the dominant *bla*_OXA-23_ gene were primarily associated with two major plasmid groups as follows: replicon-type (147/247, 59.51%) and other (38/247, 15.38%) plasmids. A smaller proportion of *bla*_OXA-23_ isolates showed association with multi-marker combinations, including replicon-type + other plasmids, pRAY + other plasmids, and replicon-type + pRAY + other plasmids. Although the *bla*_NDM-1_ gene was predominantly associated with a combination of replicon-type and other plasmids (4/8, 50%), a minor association with replicon-type plasmids alone was also observed. Specific co-localization patterns were critical; the *bla*_OXA-23_ + *bla*_OXA-58_ combination was exclusively linked to a multi-marker profile that included the pRAY plasmid. Furthermore, the co-presence of bla_OXA-23_ and *bla*_PER-7_ was uniquely associated with the pRAY plasmid as a single marker.

### 2.7. Comprehensive Comparative Genomics

**Figure 6:**
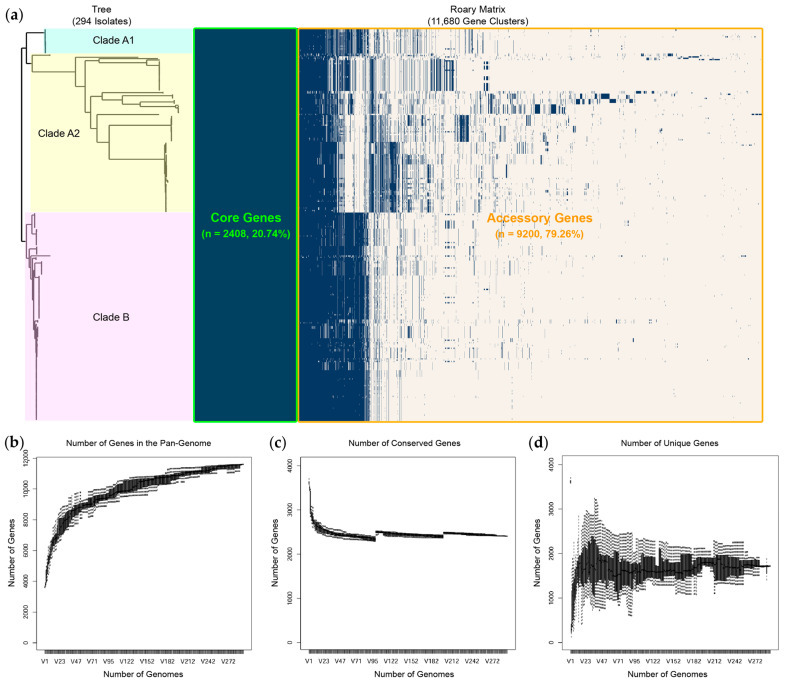
Pan-genome profiles against a core single-nucleotide polymorphism (SNP)-based phylogenetic tree of all carbapenem-resistant *Acinetobacter baumannii* (CRAB) clinical isolates (**a**). The number of genes in the pan-genome (**b**), the number of conserved genes (**c**), and the number of unique genes (**d**). The diagrams were generated using Roary v3.13.0.

A comprehensive comparative genomic analysis was conducted to understand the genomic epidemiology of all 294 CRAB clinical isolates. The pan-genome analysis revealed that, in 11,608 pan genes, 9200 (79.26%) and 2408 (20.74%) were classified as accessory and core genes, respectively (Figure 6a). Besides the high proportion of accessory genes, their distribution was consistent with the clade structure observed in the core SNP phylogenetic tree. As shown in Figure 6b, the total number of genes in the pan-genome increased with the addition of each genome, reaching approximately 11,500 genes. Figure 6c illustrates the number of conserved (core) genes that remained stable across all genomes, while Figure 6d shows the number of unique genes contributed by individual genomes, which ranged from approximately 1000 to 2500 genes.

**Figure 7:**
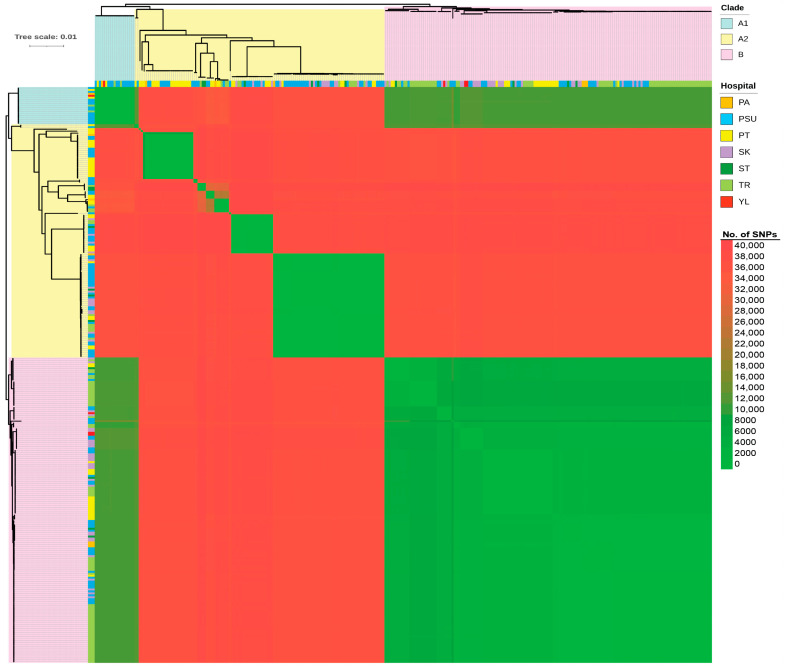
Heatmap of pairwise single-nucleotide polymorphism (SNP) distances against the core SNP-based phylogenetic tree of all carbapenem-resistant *Acinetobacter baumannii* (CRAB) clinical isolates. The heatmap and tree were generated using Microsoft Excel and FastTree v2.1.11, respectively. PA, Pattani Hospital; PSU, Songklanagarind Hospital; PT, Phatthalung Hospital; SK, Songkhla Hospital; ST, Satun Hospital; TR, Trang Hospital; YL, Yala Hospital.

**Figure 8:**
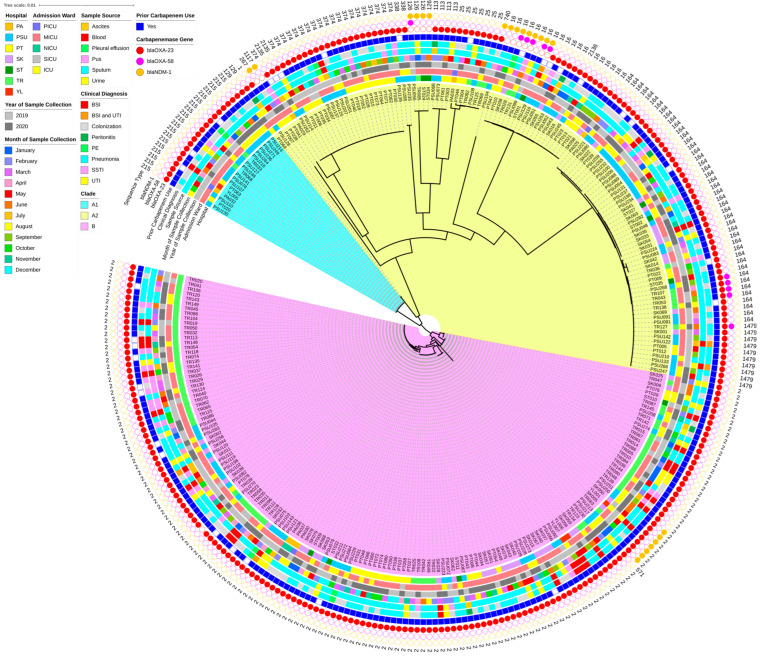
A circular phylogenetic tree constructed from single-nucleotide polymorphism (SNP) sites among carbapenem-resistant *Acinetobacter baumannii* (CRAB) clinical isolates annotated with their metadata (including hospitals, admission wards, sample sources, clinical diagnosis, prior carbapenem use, time of bacterial collection, and the presence of carbapenemase genes). The tree was generated using FastTree v2.1.11 and visualized alongside clinical and genomic metadata using iTOL v5. PA, Pattani Hospital; PSU, Songklanagarind Hospital; PT, Phatthalung Hospital; SK, Songkhla Hospital; ST, Satun Hospital; TR, Trang Hospital; YL, Yala Hospital; PICU, pediatric intensive care unit; MICU, medical intensive care unit; NICU, neonatal intensive care unit; SICU, surgical intensive care unit; ICU, intensive care unit; BSI, bloodstream infection; UTI, urinary tract infection; SSTI, skin/soft tissue infection; PE, pleural effusion.

Figure 7 presents a heatmap of pairwise SNP distances derived from the core gene alignment, along with the corresponding core SNP-based phylogeny. Meanwhile, Figure 8 shows a circular-core SNP-based phylogeny annotated with metadata. The number of SNPs ranged from 0 to nearly 40,000. Although the distribution showed clear clustering according to distinct STs, some STs were grouped within clusters of other STs. The core SNP-based phylogenetic tree revealed two main polyphyletic clades (A and B), with clade A being further subdivided into two subclades (A1 and A2). Clades A1 and B contained ST215 and ST2 isolates, respectively, whereas clade A2 contained multiple STs. Most isolates (41.29%) obtained from patients admitted to Trang Hospital were clustered within the large clade B (ST2 lineage), whereas relatively few isolates were observed in clades A1 (10%) and A2 (7.45%). A high proportion of the isolates (65%) from Songklanagarind Hospital were grouped within clade A2, comprising multiple STs (except for ST2, ST215, and ST571). Isolates from Phatthalung Hospital were the most diverse population and were well represented across all three clades. Although a small proportion of the isolates was obtained from patients admitted to Pattani and Yala Hospitals, they were distributed across all three clades. Sample sources were associated with clinical diagnoses; however, a large number of isolates were recovered from the sputum of patients with CRAB-colonization. Isolates from patients with BSI were mostly associated with clade B. Similarly, isolates from patients with SSTI who were admitted to Trang Hospital were predominantly localized to clade B.

Remarkably, ST571 isolate (PSU217) was clustered with ST2 isolates in clade B. ST571 isolate showed the highest number of SNPs (indicated by the most intense red colouration) in the pairwise SNP comparison among ST2 isolates within clade B. Additionally, ST2136 isolate (PSU043) was grouped with ST16 isolates within a subclade of clade A2. Our study demonstrated that the isolates were dispersed across hospitals, admission wards, sample sources, clinical diagnoses, collection times, and carbapenemase gene types, with no distinct clustering. Co-occurrence of *bla*_OXA-23_ and *bla*_NDM-1_ was observed in an ST740 isolate and some ST2 isolates (*n* = 7), which were collected from patients at different hospitals over various years. Co-harboring of *bla*_OXA-58_ and *bla*_NDM-1_ was detected in one ST126 isolate and five ST16 isolates, respectively. In contrast, *bla*_OXA-23_ and *bla*_OXA-58_ were identified in four ST164 isolates and one ST1479 isolate, respectively, all of which were obtained from the sputum of patients in medical intensive care units. The isolates belonging to ST267 and ST1117 harbored only *bla*_NDM-1_. Interestingly, almost all the isolates possessed at least one carbapenemase gene; however, the PSU270 isolate belonging to ST2 (clade B) lacked any detectable carbapenemase genes.

**Figure 9:**
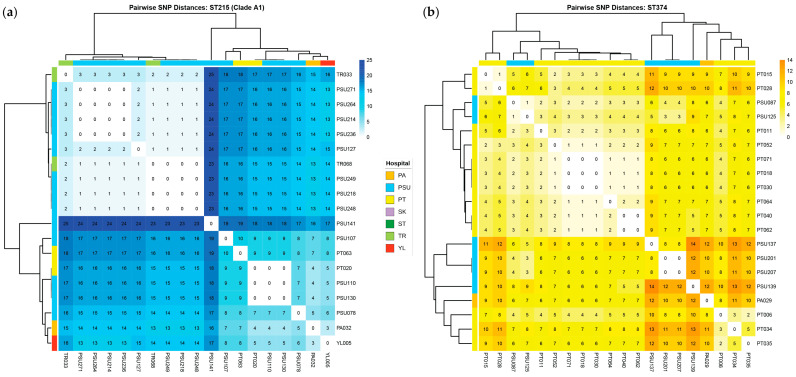
Heatmaps of pairwise single-nucleotide polymorphism (SNP) distances against the core SNP-based phylogenetic tree of specific clusters: ST215 (**a**) and ST374 (**b**). The heatmaps were generated using the pheatmap package in R. PA, Pattani Hospital; PSU, Songklanagarind Hospital; PT, Phatthalung Hospital; SK, Songkhla Hospital; ST, Satun Hospital; TR, Trang Hospital; YL, Yala Hospital.

Pairwise SNP distances within specific clusters were analyzed using established CRAB SNP thresholds to infer transmission events. Clusters with <21 SNP differences were considered to be outbreak-associated, whereas 0–10 SNP differences indicated highly likely direct transmission [15,16]. Figure 9 shows the complex landscape of the local outbreaks and their regional dissemination. Analysis of clade A1 (ST215) provided evidence of both explosive single-center outbreaks and broader regional persistence (Figure 9a). A primary cluster of identical isolates (0 SNP) was identified among PSU271, PSU264, PSU214, and PSU236. Evidence of regional spread was further supported by the genetic similarity observed between the TR068 isolate from Trang Hospital and several other isolates from Songklanagarind Hospital (PSU249, PSU218, and PSU248). Although many isolates remained tightly clustered, the presence of outliers, such as PSU141, resulted in 19–25 SNPs that were distant from the main group. Similarly, high-resolution SNP analysis of ST374 isolates identified a significant direct transmission cluster at Phatthalung Hospital, involving PT071, PT018, and PT030, which were genetically identical with 0 SNP differences (Figure 9b). Furthermore, isolates from Phatthalung Hospital (PT040 and PT062) were within the 0–2 SNP range of isolates from Songklanagarind Hospital (PSU087 and PSU125), falling well below the established 10-SNP threshold for highly likely direct transmission.

## 3. Discussion

Surveillance of clinical CRAB across seven hospitals in Thailand revealed a high, persistent burden of this pathogen within the regional healthcare ecosystem. The endemic nature of CRAB was highlighted by its continuous isolation over the 24-month study period from 2019 to 2020, with no significant seasonal gaps observed across the major contributing centers. Notably, the disproportionate burden observed at Songklanagarind and Trang Hospitals suggests that these facilities serve as important regional reservoirs for CRAB. As a major tertiary referral center in Southern Thailand, the high isolation rate at Songklanagarind Hospital likely reflects its complex patient population and the high frequency of interhospital transfers, which are known drivers of multidrug-resistant organism dissemination. The isolation timeline of CRAB isolates displayed a high-resolution view of transmission dynamics, with distinct temporal clusters and multiple isolates recovered within narrow 10-day intervals, strongly suggesting localized outbreaks or clonal expansion within specific wards. Notably, the increase in isolates at Phatthalung Hospital in 2020, despite an overall downward trend in other centers, warrants further investigation into local infection control practices or potential changes in antibiotic stewardship during that period. Conversely, the absence of CRAB isolates from Yala Hospital in 2020, following their presence in 2019, indicates the success of targeted intervention strategies.

WGS analysis showed that all CRAB clinical isolates in this study carried a broad set of VAGs, suggesting a strong potential for persistence and pathogenicity. The universal presence of efflux pump and penicillin-binding protein genes highlights the core mechanisms of antimicrobial resistance, whereas biofilm-associated genes indicate an enhanced ability to form biofilms and withstand host defenses [17,18]. Complete siderophore clusters further underscore the conserved capacity for iron acquisition, which is a critical factor in infection. Lipopolysaccharide and lipid A biosynthesis genes and other virulence determinants reinforce the intrinsic virulence potential of these isolates. These findings demonstrate that CRAB in this region maintains a broad genetic arsenal of resistance and virulence factors. Importantly, the ubiquitous presence of VAGs across all isolates suggests a highly conserved core virulome within the CRAB population in Southern Thailand. This homogeneity indicates that the regional risk is driven more by the widespread dissemination of well-equipped lineages rather than the emergence of specific hypervirulent sub-clones.

MST analysis revealed three CCs based on the STs identified in all 294 CRAB clinical isolates. CC1, centered on ST374, comprised several closely related STs, indicating the clonal expansion of a distinct lineage [19]. The predominance of respiratory isolates suggests an important role in hospital-associated pneumonia, although its smaller size and diversity compared with CC2 indicate a more limited epidemiological spread. ST2 isolates were clustered together with ST129 and ST740 isolates in CC2, which is consistent with the IC2 lineage, a globally disseminated high-risk clone strongly associated with carbapenem resistance and hospital-acquired infections [20,21]. ST2, the predominant sequence type, likely represents the founder genotype within CC2, whereas ST129 and ST740 appear to be closely related variants that emerged through local microevolution. The recovery of CC2 isolates from various clinical specimens, particularly respiratory samples, and from invasive sources, underscores the clinical adaptability and epidemiological success of this lineage. CC3 consisted of a small cluster centered on ST215 with limited clonal diversity and fewer isolates, suggesting sporadic occurrence or restricted transmission. Collectively, MST demonstrated the population structure of the CRAB clinical isolates, with CC2 representing a dominant endemic IC2 lineage, CC1 indicating an additional locally expanding clone, and CC3 reflecting a minor or sporadically distributed lineage.

The association between resistance and virulence scores revealed the distribution of CRAB STs into four groups based on the selected thresholds. Most STs (*n* = 8) possessed high resistance and virulence traits, indicating a critical combination of high antimicrobial resistance and virulence potential. ST2 isolates, which are the most commonly identified STs, exhibited relatively high median resistance and the highest virulence scores. These findings align with previous reports showing that ST2 is the predominant global clone associated with extensive drug resistance, prolonged environmental persistence, and high clinical prevalence [22,23]. The co-occurrence of multiple resistance determinants and VAGs, such as those encoding biofilm-associated proteins, chaperone-usher fimbriae, quorum sensing, and other virulence factors (iron uptake systems), may underlie the success of ST2 in hospital environments [24]. In addition to ST2 isolates, ST1117 isolates exhibited the highest median virulence scores (approximately 7) and relatively high resistance (approximately 5). Given the limited data on ST1117 in CRAB, we hypothesized that this lineage may have acquired mobile genetic elements or resistance islands co-carrying antimicrobial resistance and virulence determinants. Selective pressure within hospital environments could have favored the emergence of such a dual-advantage clone capable of persistence, resistance, and pathogenicity. Compared with the typical resistance–virulence trade-off observed in some lineages, ST1117 may retain both traits without an apparent fitness cost. Other STs within this high-resistance, high-virulence group, particularly ST16, ST25, ST126, and ST21, also merit attention, as some have been sporadically reported in clinical isolates with multidrug resistance characteristics and may represent emerging or regionally adapted lineages [22,25,26,27]. Six STs exhibited high resistance and low virulence, whereas three STs displayed the opposite trend (low resistance but high virulence), suggesting possible trade-offs between antimicrobial resistance and virulence among some lineages. Only ST2135 isolates were categorized in the quadrant of low-resistance and low-virulence features, indicating a relatively less-adapted CRAB lineage with limited clinical impact. Importantly, the resistance–virulence scores calculated in this study did not correlate significantly with clinical outcomes. The designation of high-risk STs refers to their epidemiological fitness—specifically, their ability to maintain a broad antimicrobial resistance profile alongside a stable virulence repertoire. These characteristics facilitate their persistence within the hospital environment and their successful dissemination across the regional network, representing a significant public health challenge regardless of individual patient outcomes.

According to the Sankey diagrams showing the associations between clinical and genomic data, Figure 5a suggests that, while various genetic lineages can cause severe manifestations, such as pneumonia or BSI, some of them typically colonize within the patients [28]. ST2 lineage’s ability to persist across multiple hospital sites suggests that it is a highly adapted, endemic, high-risk clone that poses a consistent threat to patient health throughout the region [29]. Songklanagarind Hospital is a unique epicenter of clinical complexity within the region, characterized by a high volume of severe cases and a distinct genetic landscape. While the hospital aligns with broader regional trends in pneumonia and colonization, its role as a tertiary care center is evidenced by its exclusive management of peritonitis, UTI, and concurrent BSI and UTI. This intricate clinical flow suggests that a patient population with higher acuity and significant underlying comorbidities necessitates specialized interventions that distinguish it from smaller regional facilities. Colonization acts as a central genetic hub, hosting a wide array of specialized STs that remain largely asymptomatic. However, a few selected lineages, most notably the global generalist ST2, possess the necessary virulence to breach host defenses and cause systemic diseases [30]. Furthermore, a large number of ST215 isolates colonize in the patients. These findings highlight the critical need for institutional surveillance and tailored infection control strategies that address the threat of dominant global clones and site-specific genetic variation persistence [31].

The data presented in Figure 5b provide good evidence for a primarily clonal dissemination model for carbapenem resistance. Although short-read data limit the complete de novo assembly and unambiguous typing of entire plasmids, it successfully enables the prediction of plasmid marker genes and replicon types (e.g., repAci1, p1ABSDF, and pRAY). Specifically, the model was driven by the dominance of ST2 and ST164 isolates, which correspond to the high-risk IC 2. The confluence of most STs into *bla*_OXA-23_ suggests that this resistance mechanism has recently emerged in these dominant clones or provides a superior fitness advantage that promotes its maintenance and spread across the bacterial population [32,33]. Additionally, the clear split between replicon-type and other plasmids indicated that *bla*_OXA-23_ is not strictly confined to a single well-established mobile element family. This plasticity, in which the gene is successfully harbored on both common, characterized plasmids and a diverse range of unclassified elements, enhances its potential for horizontal gene transfer (HGT) and complicates surveillance efforts [22]. The observation that less prevalent genes, such as *bla*_NDM-1_, were primarily found in minor genetically distinct STs indicates that these resistance determinants represent independent introduction events rather than successful clonal expansion within the current study environment. Importantly, these genes were mainly associated with replicon-type plasmids, reflecting different initial mobilization pathways compared with the highly successful *bla*_OXA-23_.

In the comparative genomic analysis of all 294 CRAB clinical isolates, pan-genome profiles mapped onto the core SNP phylogenetic tree revealed an open pan-genome with abundant accessory genes, shaping distinct clades. Gene distribution closely followed clade differentiation, indicating lineage-specific acquisition, whereas a conserved core set coexisted with unique strain-specific genes, underpinning the broad genetic diversity among isolates. Although the SNPs obtained from the core gene alignment enabled in-depth genomic comparisons within the species, some STs were clustered within the phylogenetic subclades of other STs. This observation indicates underlying genetic relatedness or potential limitations of ST-based discrimination [34,35]. Previous studies have reported that ST assignment using the Pasteur and Oxford schemes may not always reflect the true genomic relationships [34,35]. MLST relies on a few housekeeping genes and may not fully capture the genomic diversity of complex species with highly recombinant genomes and frequent HGTs, such as *A. baumannii*. The core SNP phylogeny combined with metadata suggested clonal heterogeneity among hospitals, with ST2 (clade B) dominating several sites (particularly Trung Hospital), whereas ST215 (clade A1) appeared sporadically. Multiple STs (clade A2) presence in most hospitals indicates ongoing diversification or numerous introduction events. The tree also revealed that a large number of isolates were collected from the sputum of patients with CRAB colonization, suggesting that the respiratory tract is a primary site for colonization. Furthermore, isolates from patients with concomitant BSI and SSTI were predominantly clustered within clade B. This indicates that ST2 isolates possess higher virulence than other STs [30,36]. The pairwise SNP distances of ST215 (clade A1) and ST374 isolates revealed both localized outbreaks and extensive regional dissemination of CRAB in Southern Thailand. Moreover, the identification of identical isolates (0 SNPs) in Songklanagarind Hospital suggests high-intensity transmission from common point sources. Critically, the negligible genetic distance (0–2 SNPs) between isolates from Phatthalung and Songklanagarind Hospitals indicates that patient or equipment transfer links these facilities into a single epidemiological network. Although active clusters drove acute outbreaks, more divergent outliers (12–25 SNPs) highlighted the long-term persistence of environmental reservoirs alongside these transmission chains.

Combined with the association between resistance and virulence (Figure 3) and the core SNP-based phylogenetic relationship (Figure 7), we found that *bla*_OXA-23_-carrying ST2135 isolates (*n* = 2), which showed low resistance and virulence, were obtained from patients at Songklanagarind Hospital over different years without prior carbapenem exposure within three months and clustered within a specific subclade of ST374. Meanwhile, the *bla*_NDM-1_-carrying ST126 (*n* = 3) and *bla*_NDM-1_- and *bla*_OXA-58_-carrying ST126 (*n* = 1) isolates, which exhibited high resistance and virulence, were collected from patients at three distinct hospitals and in different years with prior carbapenem exposure within three months and clustered within their specific subclade. These findings align with the results from previous studies showing that such isolates can cause severe infections, highlighting that high resistance and virulence may co-occur in clinical settings (e.g., a recent outbreak of multidrug-resistant *A. baumannii* ST126 carrying *bla*_NDM-1_ and *bla*_OXA-58_ located on the plasmid) [25]. Importantly, none of the carbapenemase genes were detected in one of the ST2 isolates (PSU270), indicating a distinct resistance profile compared with other CRAB clinical isolates. However, other class D oxacillinase genes were observed in this isolate, suggesting that its carbapenem resistance is mediated by oxacillinase-like enzyme production in combination with alternative mechanisms, including porin loss (e.g., CarO and OprD-like proteins) or efflux pump overexpression (AdeABC, AdeIJK, and AdeFGH systems), altered PBPs, and/or increased expression of AmpC β-lactamases [4,37].

## 4. Materials and Methods

### 4.1. Carbapenem-Resistant Acinetobacter baumannii Clinical Isolates

Recently sequenced CRAB genomes deposited in the NCBI under BioProject number PRJNA1154797 (*n* = 70), along with our previously published genomes under BioProject numbers PRJNA752484 (*n* = 219) [38] and PRJNA940623 (*n* = 5) [39], were included in this study. All 294 non-repetitive CRAB clinical isolates were collected as part of a surveillance program that monitored carbapenem-resistant Gram-negative pathogens within a hospital network in the lower part of southern Thailand from 2019 to 2020. These isolates were obtained from patients admitted to the following seven hospitals: Satun (*n* = 10), Trang (*n* = 78), Phatthalung (*n* = 53), Songklanagarind (*n* = 99), Songkhla (*n* = 43), Pattani (*n* = 7), and Yala (*n* = 4) Hospitals.

### 4.2. Genomic DNA Extraction and Whole-Genome Sequencing

The genomic DNA of the 70 CRAB clinical isolates sequenced in this study was extracted using a GF-1 Bacterial DNA Extraction Kit (Selangor, Malaysia), following the manufacturer’s instructions. The concentration and purity of the extracted DNA samples were verified using a NanoDrop 2000/2000c spectrophotometer (Thermo Scientific, Wilmington, NC, USA), and degradation was observed using agarose gel electrophoresis. Qualified DNA samples were subjected to short-read WGS using the MGISEQ-2000 sequencer (MGI Tech Co., Ltd., Shenzhen, China) under a 150-bp paired-end reads platform at the Beijing Genomic Institute (BGI), China.

### 4.3. De Novo Assembly, Species Confirmation, and Genome Annotation

De novo assembly, species confirmation, and genome annotation were performed for 294 CRAB genomes. The reads were de novo assembled using SPAdes v3.12 [40]. Additionally, the assembled genomes’ quality and completeness were assessed using QUAST v4.0 [41] and BUSCO v5.2.2 [42], respectively. *A. baumannii* species was confirmed by comparing with reference genomes of all *Acinetobacter* species using FastANI v1.32 [43]. Subsequently, the qualified assembled genomes were annotated using Prokka v1.12 [44].

### 4.4. Downstream Analysis

Downstream analyses were performed on all 294 CRAB genomes. STs and ARGs were identified using StarAMR v0.10.0 against the PubMLST and ResFinder databases [45]. Genomes with unidentified STs were submitted to the PubMLST database to define new STs. An MST was created using PHYLOViZ v2.0 [46]. To investigate the virulence of these pathogens, VAGs were analyzed using BLASTN v2.16.0 against reference sequences of the most frequent VAGs in *A. baumanii* [13,38]. PMGs were predicted using BLASTN against reference sequences of the most frequent plasmid replicase genes in *A. baumanii* to investigate HGT [13,38]. The association between antimicrobial resistance and virulence determinants across the identified STs was examined by scoring the presence of selected ARGs and VAGs. A quadrant scatter plot was generated using R version 4.4.2. Associations among hospitals, diagnoses, and STs, as well as among STs, carbapenem resistance-associated genes, and predicted PMGs were also analyzed. Sankey diagrams were created using R version 4.4.2, and pan-genome profiles were investigated employing Roary v3.13.0 [47]. Pairwise SNPs were called from the core gene alignment using SNP-dists v0.8.2 for calculating the pairwise SNP distance matrix (https://github.com/tseemann/snp-dists?tab=readme-ov-file (accessed on 26 August 2025)) and SNP-sites v2.4.1 for extracting the SNP sites [48]. A phylogenetic tree was constructed based on core SNP analysis using FastTree v2.1.11 with the maximum-likelihood method and 1000 bootstrap replicates [49]. The circular tree was generated using iTOL v5 [50].

## 5. Conclusions

This study revealed a structured and diverse population of CRAB clinical isolates driven by both globally disseminated high-risk clones and locally evolving lineages. All isolates carried a conserved repertoire of ARGs and VAGs, underscoring their intrinsic capacity for persistence and pathogenicity in hospital environments. Population analyses identified three CCs, with CC2 representing the dominant IC2 lineage centered on ST2. This lineage, particularly ST2 isolates, demonstrated extensive diversification, broad clinical involvement, and highly combined antimicrobial resistance and virulence traits, confirming its central role in regional CRAB epidemiology. CC1 reflected localized clonal expansion, whereas CC3 appeared sporadic. Core SNP phylogenetic analysis identified three clades, with ST2 isolates predominantly clustering in the large clade B. The ST571 isolate also clustered within clade B, despite showing a higher SNP divergence than ST2 isolates. The observed SNP divergence supports ongoing microevolution within clade B and highlights the higher discriminatory power of core SNP analysis over MLST for resolving population structure. Additionally, the core SNP phylogeny combined with the metadata displayed clonal heterogeneity across hospitals. A large proportion of isolates were recovered from the sputum of patients with CRAB colonization, indicating that the respiratory tract is a major reservoir for colonization. Pneumonia-associated isolates were distributed across multiple hospitals, whereas BSI- and SSTI-associated isolates clustered predominantly within clade B, suggesting an increased invasive potential of ST2. The integration of genomic and clinical data highlighted the respiratory tract as a major reservoir of CRAB diversity and emphasized the dynamic transition from colonization to invasive infection. Collectively, these findings underscore the need for sustained genomic surveillance and tailored infection control strategies that target both dominant global clones and persistent local variants.

## Figures and Tables

**Table 1:** Patients’ clinical characteristics.

Characteristics	Patients (N = 294)
Demographics	
Age, Median [IQR] (years)	52 [34, 66]
Male	172 (58.50%)
Female	122 (41.50%)
Underlying diseases	
Diabetes mellitus	131 (44.56%)
Hypertension	98 (33.33%)
Chronic kidney disease	75 (25.51%)
Cerebrovascular disease	52 (17.69%)
Coronary artery disease	56 (19.05%)
Pulmonary disease	38 (12.93%)
CCI score, Median [IQR]	7 [5, 8]
Prior antibiotic use within 3 months	
Ceftriaxone	174 (59.18%)
Ceftazidime	38 (12.93%)
Imipenem	140 (47.62%)
Meropenem	219 (74.49%)
Ertapenem	32 (10.88%)
Piperacillin/tazobactam	164 (55.78%)
Aminoglycosides (amikacin or gentamycin)	32 (10.88%)
Fluoroquinolones (levofloxacin or ciprofloxacin)	98 (33.33%)
Others: azithromycin, colistin, tigecycline, cefoperazone/sulbactam, or antifungal agents	59 (20.07%)
Clinical diagnosis	
Infection	
Bloodstream infection	25 (8.50%)
Bloodstream infection and urinary tract infection	5 (1.70%)
Peritonitis	6 (2.04%)
Pleural effusion	5 (1.70%)
Pneumonia	122 (41.50%)
Skin/soft tissue infection	19 (6.46%)
Urinary tract infection	24 (8.16%)
Colonization	88 (29.93%)
APACHE II score, Median [IQR]	16 [12, 16]
Length of hospital stay, Median [IQR]	12 [9, 19]

IQR, interquartile range; CCI, Commodity Channel Index; APACHE II, Acute Physiology and Chronic Health Evaluation II.

**Table 2:** Antimicrobial susceptibility testing of all carbapenem-resistant *Acinetobacter baumannii* (CRAB) clinical isolates.

Antimicrobial Agents	Interpretation	Hospitals, n (%)						
		PA (*n* = 7)	PSU (*n* = 99)	PT (*n* = 53)	SK (*n* = 43)	ST (*n* = 10)	TR (*n* = 78)	YL (*n* = 4)
IPM	Susceptible	0 (0)	6 (6.06)	2 (3.77)	0 (0)	1 (10)	1 (1.28)	0 (0)
	Resistant	7 (100)	93 (93.94)	51 (96.23)	43 (100)	9 (90)	77 (98.72)	4 (100)
MEM	Susceptible	0 (0)	4 (4.04)	3 (5.66)	1 (2.33)	1 (10)	2 (2.56)	0 (0)
	Resistant	7 (100)	95 (95.96)	50 (94.34)	42 (97.67)	9 (90)	76 (97.44)	4 (100)
CAZ	Susceptible	0 (0)	4 (4.04)	1 (1.89)	1 (2.33)	0 (0)	1 (1.28)	0 (0)
	Resistant	7 (100)	95 (95.96)	52 (98.11)	42 (97.67)	10 (100)	77 (98.72)	4 (100)
CEP/SUL	Susceptible	0 (0)	13 (13.13)	8 (15.09)	5 (11.63)	1 (10)	6 (7.69)	1 (25)
	Resistant	7 (100)	86 (86.87)	45 (84.91)	38 (88.37)	9 (90)	72 (92.31)	3 (75)
AMK	Susceptible	0 (0)	4 (4.04)	2 (3.77)	1 (2.33)	1 (10)	1 (1.28)	0 (0)
	Resistant	7 (100)	95 (95.96)	51 (96.23)	42 (97.67)	9 (90)	77 (98.72)	4 (100)
GEN	Susceptible	0 (0)	4 (4.04)	2 (3.77)	1 (2.33)	0 (0)	2 (2.56)	0 (0)
	Resistant	7 (100)	95 (95.96)	51 (96.23)	42 (97.67)	10 (100)	76 (97.44)	4 (100)
CIP	Susceptible	0 (0)	3 (3.03)	1 (1.89)	1 (2.33)	1 (10)	0 (0)	0 (0)
	Resistant	7 (100)	96 (96.97)	52 (98.11)	42 (97.67)	9 (90)	78 (100)	4 (100)
LVX	Susceptible	0 (0)	3 (3.03)	1 (1.89)	1 (2.33)	0 (0)	0 (0)	1 (25)
	Resistant	7 (100)	96 (96.97)	52 (98.11)	42 (97.67)	10 (100)	78 (100)	3 (75)
CST	Susceptible	7 (100)	91 (91.92)	50 (94.34)	40 (93.02)	10 (100)	76 (97.44)	4 (100)
	Resistant	0 (0)	8 (8.08)	3 (5.66)	3 (6.98)	0 (0)	2 (2.56)	0 (0)
TMP/SMX	Susceptible	0 (0)	9 (9.09)	8 (15.09)	9 (20.93)	0 (0)	9 (11.54)	1 (25)
	Resistant	7 (100)	90 (90.91)	45 (84.91)	34 (79.07)	10 (100)	69 (88.46)	3 (75)
TIG	Susceptible	7 (100)	81 (81.82)	48 (90.57)	41 (95.35)	10 (100)	75 (96.15)	4 (100)
	Resistant	0 (0)	18 (18.18)	5 (9.43)	2 (4.65)	0 (0)	3 (3.85)	0 (0)

PA, Pattani Hospital; PSU, Songklanagarind Hospital; PT, Phatthalung Hospital; SK, Songkhla Hospital; ST, Satun Hospital; TR, Trang Hospital; YL, Yala Hospital; IPM, imipenem; MEM, meropenem; CAZ, ceftazidime; CEP/SUL, cefoperazone/sulbactam; AMK, amikacin; GEN, gentamicin; CIP, ciprofloxacin; LVX, levofloxacin; CST, colistin; TMP/SMX, trimethoprim/sulfamethoxazole (co-trimoxazole); TIG, tigecycline.

## Data Availability

This Whole Genome Shotgun project has been deposited in DDBJ/ENA/GenBank under BioProject number PRJNA1154797 and BioSample numbers SAMN43433587–SAMN43433656.

## Supplementary Materials

The following supporting information can be downloaded at: https://www.mdpi.com/article/10.3390/antibiotics15020133/s1, Table S1: Detailed clinical information on CRAB clinical isolates; Table S2: Antimicrobial susceptibility patterns in all CRAB clinical isolates; Table S3: Genome quality reports and NCBI information of all CRAB clinical isolates; Table S4: The results of multilocus sequence typing (MLST) using the Pasteur scheme.

## Abbreviations

The following abbreviations are used in this manuscript:

BSI	Bloodstream infection
SSTI	Skin/soft tissue infection
SNP	Single-nucleotide polymorphism
CRAB	Carbapenem-resistant *Acinetobacter baumannii*
PBP	Penicillin-binding protein
IC2	International Clone 2
GC	Global Clonal
GC2	Global Clonal Group 2
HGT	Horizontal gene transfer
ESBL	Extended-spectrum β-lactamase
VAGs	Virulence-associated genes
ARGs	Antimicrobial resistance genes
PMGs	Plasmid marker genes
CC	Clonal complex
MST	Minimum spanning tree
CCI	Commodity Channel Index
APACHE II	Acute Physiology and Chronic Health Evaluation II
IQR	Interquartile range
IS	Insertion sequence
WGS	Whole-genome sequencing
MLST	Multilocus sequence typing
